# Important Bioactive Molecules of Erythrocytes in Colorectal Cancer Patients After Colectomy

**DOI:** 10.2174/1874104500802010006

**Published:** 2008-02-27

**Authors:** Anna Blázovics, Ágnes Szilvás, György Székely, Enikő Tordai, Edit Székely, Gábor Czabai, Zsolt Pallai, Éva Sárdi

**Affiliations:** 1 II. Department of Medicine, Semmelweis University, Budapest; 2 Saint John Hospital, Budapest, Hungary; 3 Department of Genetics and Plant Breeding, Corvinus University of Budapest, Budapest, Hungary; 4 Central Hospital of the Hungarian State Railways, Budapest, Hungary; 5 Diachem Ltd., Budapest, Hungary

**Keywords:** Colorectal cancer, HCHO, protoporphyrin, redox homeostasis.

## Abstract

Formaldehyde (HCHO) and protoporphyrin, are in connection with redox homeostasis. Data show the importance of HCHO in proliferative as well as in apoptotic processes. Free protoporphyrin can be detected near the Zn-protoporphyrin in cancer and it has pro- and antioxidant forms depending on concentrations. The aim was to determine the amount of HCHO and protoporphyrin concentrations of erythrocytes in colorectal cancer after colectomy and to estimate redox homeostasis. Total 32 adult patients after 5-10 years of colectomy and 9 healthy volunteers were drawn into this study. Tumor markers, redox parameters, HbA1c, HCHO and protoporphyrin concentrations were measured. Erytrocyte HCHO was significantly lower in colectomysed patients, than in controls. Protoporphyrin concentration was low in patients, but in metastasis its concentration was significant. HbA1c correlated significantly with free radicals and decreased the antioxidant status of erythrocytes. HCHO and protoporphyrin concentrations of erythrocytes and the total scavenger capacity are very important indexes in cancer.

## INTRODUCTION

Colorectal cancers are one of the most frequent types of cancers in Hungary as well as in the USA. Often the diseases are observed only in Dukes B and C stages or later stages and tumor markers can show false negative results.

The resection of cancerous tissues does not prevent the eventual recurrence of the cancer therefore it is very important to check for recidives or metastasis. The survival significantly depends on the early recognition of metastases.

HCHO is genotoxic and carcinogen. It can be found in animal tissues in a special bounded form, mainly in hydroxymethyl form. Endogenous HCHO is produced partly by enzymatic demethylation of different N-, S-, and O- methylated compounds [1, 2]. S-adenosyl-methionine is an important methyl donor in several biological transmethylation reactions. The product of the S-adenosyl-homocysteine inhibits the transmethylation process because this molecule is hydrolysed to adenosine and L-homocysteine by the action of S-adenosyl-homocysteine hydrolase [3]. The accumulation of homocysteine leads to increased cellular oxidative stress [4]. DNA methylation typically occurs at the cytosine – phosphate – guanine site. This methylation results in the conversion of the cytosine to 5-methyl-cytosine. As the result of this methylation in the conversion of the cytosine is converted into 5-methyl-cytosine. This reaction is catalysed by DNA methyltransferase. The methylation stage of this region can  have a major    impact  on  gene   functions  [5].  Since the endogenous transmethylation processes occur *via *HCHO, this molecule can be considered as an ancient and basic compound of life systems [1, 6, 7]. Some quaternary ammonium compounds, such as N^ε^–trimethyl-L-lysine or choline are potential HCHO generators as well [2]. It was also verified, that arginine (38), methionine (65) and lysine (72) near the methionine (80) are methylated and coordinated towards the central iron of heme [8].

The protoporphyrin IX. heterocyclic macromolecule is formed by the activity of protoporphyrinogen oxidase, and this molecule will be the substrate of ferrochelatase to form heme in the process of hemoglobin biosynthesis. It is well known that hemoglobin biosynthesis occurs partly in cytosol and the mitochondrion, and than heme and globine are connected with each other in the cytosol.

Porphyrins combine readily with metals. Hemoglobin contains iron or zinc. Porphyrines are also very ancient molecules in the flora and fauna [9, 10].

Iron-porphyrine proteins represent an important group of redoxy enzymes, such as catalase, peroxidase, cytochrome c and so on. Cytochrome c methylation processes were described in arginine, methionine and lysine by specific methyltransferases [11]. Biological transmethylation - in which HCHO cannot be released - is very important for its structural and functional aspects [12-16]. For example both L-arginine supplementation and deprivation influence cell proliferation. The diverse biological effects of L-arginine and its methylated derivatives encourage further experiments [17]. It is also described, that HCHO can react with nucleofil agents e.g. glutathione. ^1^O_2 _is produced in the reaction of HCHO and H_2_O_2 _[18].

The captured formaldehyde is originated from dynamic methylation and demethylation processes in which this simplest aliphatic aldehyde is bound in form of highly reactive hydroxymethyl groups. The basis of the formaldemethone formation is that the small quantity of HCHO in equilibrium with the hydroxymethyl groups will react with dimedone to form formaldemethone. This will disturb the equilibrium and more HCHO will be formed, which, in turn, will react with the dimedone [2].

In case of colorectal tumors both primer and secunder preventions have very important roles. In previous papers, we gave account that the erythrocyte redox state is a very good indicator of the severity of different gastrointestinal diseases [19-22]. We wanted to know, whether the recent developed simple routine methods were suitable to examine the recidive or metastasis of colectomysed patients. The question is whether the operation and its follow up chemotherapy and radiotherapy will still be effective in later years? The further aims of this study are to determine the HCHO and protoporphyrin concentrations of erythrocytes in colorectal cancer after colectomy and to examine the connection of molecules such as free radicals, HCHO and protoporphyrin, to establish the concentration ratios of the active molecules in erythrocyte of colectomised patients, and to explain their roles in the redox homeostasis.

## MATERIALS AND METHODS

### Materials.

1,1-diphenyl-2-picrylhydrasyl stable radical, luminol, hydrogen peroxide and microperoxidase were obtained from SIGMA (St. Louis). Tumor marker CEA, CA 19-9, AFP and PSA kits (LIA-mAT immunoluminometry) were obtained from LIA-mAT (Budapest) and HbA1c-kit from BioRad (Budapest). CRP (CRP/AUT-000) was obtained from Diagnosticum Ltd. GSHPx and SOD kits were purchased from RANDOX (England). All other reagents in analytical grade were purchased from Reanal (Budapest).

### Patients.

Caucasian volunteers, 32 adult patients (18 male and 14 female) (aged: 60,4 ± 8,8 years) after 5-10 years of their colectomy and chemotherapy (N=32) and radiotherapy (N=8) and 9 healthy volunteers (4 male and 5 female) (aged: 55,8±9,7 years) were drawn into this study. Nine control patients and 20 Dukes B and 5 Dukes C patients were included in the study, all of them being free of complaints. They underwent routine examinations as well as 3D abdominal ultrasound examinations. Patients with positive routine or 3D ultrasound examinations underwent further examinations (endoscopy and CT).

Tumor markers (CEA, CA 19-9, AFP / Berthold Lumat 9501 manual instrument), CRP, redox parameters, HbA1c (Variant II. HPLC method), HCHO and protoporphyrin concentrations in the erythrocyte were measured with routine laboratory parameters together. Patients with Dukes B and C after operation received same recommended standard therapy.

Permission number: TUKEB 167/1997, 15/2004 and IKEB 3944/2004.

## SEPARATION OF PLASMA AND ERYTHROCYTES

Plasma and erythrocyte were separated using the standard methods. The hemoglobin content was adjusted to 10 g% uniformly for the measurements.

### OPLC analysis.

Totality of molecular participants in transmethylating processes are not known, although the quantity of bounded HCHO could be detected in dimedon adduct form (after 24 hours) analytically.

The formaldehyde in dimedone adduct form (formaldemethone) was identified and determined by chromarographic methods using an authentic substance [23]. The samples were treated with dimedone solution (0.07% dimedone in methanol) (e.g. 0.7 cm^3^ sample / 0.7 cm^3^ of dimedone solution). This suspension was centrifuged at 1500 g for 10 minutes at 4^0^C. The clear supernatants were used to chromatographic separations [1, 23].

The chromatographyc separations were carried out on TLC silica gel 60F_254_ precoated chromatoplates (Merck Co., Germany) using chloroform - methylenechloride mixture (35/65, v/v) for formaldemethone determination. Samples were applied with a NANOMAT sample applicator (CAMAG, Switzerland). Calibration curves were prepared using an authentic substance. For densitometric determination a Shimadzu CS-930 TLC/HPTLC scanner (Shimadzu Co., Japan), λ=265 nm was used.

### Redox-state measurements.

The reducing-power of the sample was determined at 700 nm according to Oyaizu [24] based on the chemical reaction Fe[III]→Fe[II]. Increased absorbance indicated increased reducing-power, which was also expressed as an ascorbic acid equivalent (mmol/L eqAS).

The hydrogen-donating ability of the sample was estimated in the presence of 1,1-diphenyl-2-picrylhydrazyl radical according to Hatano *et al*. [25].

Free SH-group concentration was measured by the method of Sedlak based on the Ellmann reaction [1985]. All spectrophotometic methods were carried out with Jasco V 550 spectrophotometer [Jasco Co., Japan] [26].

A simple method of luminol-dependent chemiluminescence [H_2_O_2_/OH.-microperoxidase-luminol system] was applied to study the antioxidant protective mechanisms in colectomysed patients. Total scavenger capacity (TSC) of the plasma and erythrocytes was measured by this assay adapted to a Berthold Lumat 9501 manual instrument (Berthold GmbH, Germany) [19].

### Protoporphyrin detection.

Erythrocyte protoporphyrin IX was measured by the flourimetric method using JascoFP 6300 type fluorometer (Jasco Co, Japan). Simple micromethod was adapted for purification of protoporphyrin by Chisolm and coworkers [1975]. The excitation wavelength was at λ= 405 nm and the emission at λ= 610 nm [27].

One-way ANOVA statistical analysis was applied to evaluate the significance between patient groups.

The present table means ± S.D. Each measuring point represents five parallel data in luminol-dependent chemiluminescence experiments when c.v.% was under 5.00%. Significance level was determined at P<0.05.

## RESULTS

Table **1** shows the tumor marker parameters (CEA, CA 19-9, AFP) and CRP concentration of colectomised patients and healthy controls. CA 19-9 concentration of colectomised patients was significantly higher than that of controls, but the data were in normal range. There were no significant differences between genders in the measured parameters and therefore we did not separate the data of men from the women in the tables.

Four simple redox measurements, H-donating ability, reducing-power, free SH-group concentration and stimulated chemiluminescent intensity of plasma and erythrocytes were performed to evaluate the redox homeostasis in colectomysed patients compared to the control values. It can be established from these data after 5-10 years of colectomy, that the sick patients’ antioxidant defense system differed from the healthy patients’. Free SH-group concentration and reducing-power were found to change in a parallel way in the plasma and antiparallel with the chemiluminescence intensity of plasma. The erythrocyte total scavenger capacity of operated patients was significantly lower than that of the control group. On the basis of our present work high erythrocyte chemiluminescence intensity indicates low antioxidant capacity in colectomysed patients compared to healthy controls (Table **2**). HbA1c level correlated significantly the decreased antioxidant status of the erythrocyte. GSHPx and SOD activity were measured as well. Only the SOD activity was significantly decreased of the enzymatic antioxidant defense system.

Measurable amount of HCHO concentration of erythrocytes was also significantly lower in colectomised patients, than in healthy volunteers (Table **3**). Protoporphyrin concentration was very low in patients without metastasis and significantly higher value in colectomised patients after the Dukes C stage.

**Fig. (1):**
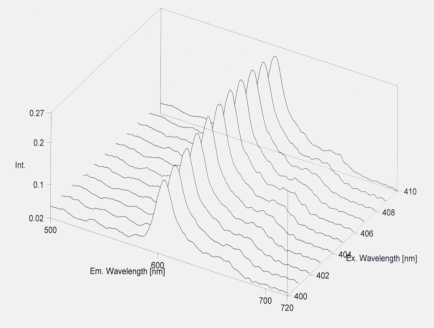
Standard curve of protoporphyrin.

**Fig. (2):**
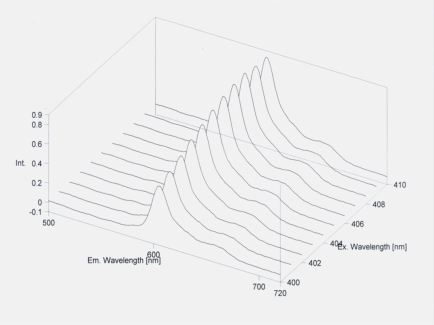
Free protoporphyrin concentration of erythrocyte in metastasis of colorectal cancer.

Seven patients were omitted from the tables, because they had protoporphyrin IX in very high concentration (600-800 nmol/L erythrocyte). The significantly lower amount of HCHO of erythrocyte was detected in five patients. It was lower than that of other operated patients and healthy controls. In these cases severe metastatic processes could be observed in cancerous patients and free protoporphyrin could be detected (Figs. **1**,**2**).

The data of seven patients were raised from the colectomised groups, because one woman was operated with mamma tumor before colectomy (HCHO cc: =2.83 x 10^-3^ μmol/mg erythrocyte), one man was treated with Xeloda because of liver metastasis (HCHO cc: 1.23 x 10^-3^ μmol/mg erythrocyte), five men had prostate problems, but only in three cases from them was tumor diagnosis established. The mean erythrocyte HCHO concentration of patients with prostate tumor was 1.53 x 10^-3^ ± 2.03 x 10^-4^ μmol/mg erythrocyte (PSA: 11.58 ± 9,02 ng/mL).

## DISCUSSION

It is well known, that in neoplasia “methylation imbalance” can be observed. Hypomethylation is accompanied by localized hypermethylation and an increase in expression of DNA methyltransferase [5, 12]. Genome-wide hypomethylation can lead to chromosome instability, and hypomethylation leads to elevated mutation rates [13]. Protoporphyrin IX generally is accumulated in more cancerous cells, than in healthy ones and autofluorescence lifetime is extended in cancer tissues. This fluorophore property of protoporphyrin is often applied in photodynamic therapy [28]. Because of all these, the idea arises, that the pool of bounded HCHO is not enough for all methylating processes in the tissues, and therefore protoporphyrin also occurs in the erythrocyte. Since methylation activity is low, hemoglobin does not form. Our results showed that in tumorous patients the protoporfirin IX – according to concentration – induces free radicals in small concentration and scavenges in higher concentration. At the same time beside the high protoporphyrin concentration the HCHO concentration was significantly low in metastatic tumorous patients. We think that erythrocyte measurements (redox homeostasis, HCHO and protoporphyrin) will bring us closer to get to know tumorous inclinations better. Determination of free protoporphyrin and bounded HCHO (mobilized methyl group) is suitable to differentiate the grade of diseases, when the results of tumor marker examinations are doubtful.

## Figures and Tables

**Table 1:** Parameters of Colectomised Patients

Groups	CEA [ng/mL] [nr: 0 - 5]	CA 19-9 [U/mL] [nr: 3.5 - 37]	AFP [U/mL] [nr: 0.5 - 11]	CRP [mg/L] [nr: < 5]
**Control [N= 9 ]**	2.8 ± 1.4	3.8 ± 2.3	4.5 ±1.9	2.34 ± 1.59
**Colectomised Patients [N=20] [Dukes B]**	1.7 ± 1.2	10.5 ± 6.7	4.1 ± 1.5	3.28 ± 2.59
**Colectomised Patients [N= 5] [Dukes C]**	1.4 ± 0.8	15.8 ± 7.1	4.7 ± 3.0	4.84 ± 6.16
**Significance: [P<0.05]**	1 *vs* 2 1 *vs* 3	1 *vs* 2 1 *vs* 3	n.s.	n.s.

**Table 2:** Parameters of Colectomised Patients

Groups	Erythrocyte CL-Intensity [RLU%]	Plasma CL-Intensity [RLU%]	Plasma H-Donor Activity [%]	Plasma Free SH-Group [mmol/L]	Plasma Reducing-Power [mmol/LeqAS]
**Control [N= 9 ]**	89.1 ± 11.4	7.9 ± 4.3	60.1 ± 7.5	0.68 ± 0.08	1.33 ± 0.29
**Colectomised Patients [N=20] [Dukes B]**	103.1 ± 14.3	10.3 ± 7.9	59.9 ± 4.4	0.55 ± 0.12	1.12 ± 0.22
**Colectomised Patients [N=5] [Dukes C]**	99.74 ± 12.33	9.6 ± 8.0	62.5 ± 4.7	0.58 ± 0.06	1.13 ± 0.17
**Significance: [P<0.05]**	1 *vs* 2	n.s.	n.s.	1 *vs* 2 1 *vs* 3	n.s.

**Table 3:** Parameters of Colectomised Patients

Groups	HbA1c [%] [nr: 3.9 - 6.1]	GSHPx [Arbitrary Unit]	SOD [Arbitrary Unit]	Erythrocyte HCHO [μmol/mg]	Proto-porphyrin IX [nmol/L erytro.]
**Control [N=9]**	5.5 ± 0.5	318.6 ± 77.4	6.6 ± 1.3	4.17 x 10^-3 ^± 1,3 x 10^-3^	n.d.
**Colectomised Patients [N=20] [Dukes B]**	5.9 ± 0.7	296.7 ± 65.0	2.3 ± 0.2	2.58 x 10^-3^ ± 3.2 x 10^-4^	n.d.
**Colectomised Patients [N=5] [Dukes C]**	6.4 ± 0.6	304.1 ± 43.2	2.6 ± 0.2	1.47 x 10^-3^ ± 2.7 x 10^-4^	397.9 ±163.2
**Significance: [P<0.05]**	1 *vs* 3	n.s.	1 *vs* 2 1 *vs* 3	1 *vs* 2 1 *vs* 3 2 vs 3	1 *vs* 3 2 *vs* 3

## ABBREVIATIONS

AFP: = Alpha fetoprotein
CA 19-9: = Carbohydrate antigen
CEA: = Carcinoembrionic antigen
CRP: = C reactive protein
HbA1c: = Oxidized hemoglobin
HCHO: = Formaldehyde
OPLC: = Over pressured layer chromatography
PSA: = Prostate specific antigen
TSC: = Total scavenger capacity
